# Surgical Interventions for the Management of Carpal Tunnel Syndrome: A Narrative Review

**DOI:** 10.7759/cureus.55593

**Published:** 2024-03-05

**Authors:** Mohummed S Alrayes, Mohammed Altawili, Mohammed H Alsaffar, Ghofran Z Alfarhan, Rahaf J Owedah, Ibtsam S Bodal, Naif Abdullah A Alshahrani, Amjad Abdulaziz M ASSIRI, Ahmad W Sindi

**Affiliations:** 1 General Surgery, King Fahad Specialist Hospital, Tabuk, SAU; 2 General Practice, Alaziziyah Primary Health Care Center, Tabuk, SAU; 3 General Practice, Abqaiq General Hospital, Buqayq, SAU; 4 Surgery, Al Iman General Hospital, Riyadh, SAU; 5 General Practice, King Fahad Hospital, Madinah, SAU; 6 General Practice, Alrayan College, Madinah, SAU; 7 General Practice, King Abdullah Hospital, Bisha, SAU; 8 Surgery, Faculty of Medicine, King Abdulaziz University, Jeddah, SAU

**Keywords:** surgical interventions, reconstruction of the transverse carpal ligament, ultrasound-guided carpal tunnel release, endoscopic carpal tunnel release, carpal tunnel syndome

## Abstract

Carpal tunnel syndrome (CTS) is a severe condition that affects the hand, causing pain, numbness, paresthesia, and autonomic dysfunction caused by increased pressure, damage, and demyelination of the median nerve in the carpal tunnel. The most effective treatment for CTS is carpal tunnel release (CTR) via transverse carpal ligament (TCL) transect. We can apply decompression through endoscopic procedures; standard open techniques and minimally invasive wrist incisions can all be used to accomplish decompression. Superior outcomes have been reported in many studies, including patient satisfaction, symptom relief, improvements in multiple assessment modality results, and fewer complications.

Soreness at the incision site, tenderness around the site of ligament release, transitory loss of motor or sensory function, and the need for a repeat operation are all postoperative consequences. There is minimal and low-quality evidence to support the effectiveness of postoperative rehabilitation, such as wrist orthoses, dressings, exercise, and ice therapy, which have benefited patients anecdotally.

## Introduction and background

Carpal tunnel syndrome (CTS) is considered the most prevalent syndrome causing focal neuropathy in the upper limbs caused by nerve entrapment [1]. Mechanical compression, damage, and increased tension in the carpal tunnel, specifically the median nerve at the wrist, are the main causes of CTS. The typical pressure of the carpal tunnel in a healthy individual ranges from 2 mmHg to 10 mmHg, but it can be up to 10 times higher in cases of CTS. The specific reason for this elevated pressure is believed to be idiopathic. Patients frequently complain of tingling sensations, numbness, and soreness in the median nerve's innervated area [2]. The prevalence of CTS ranges between 1% and 3% in a typical population [2]. Numerous risk factors, like gender, pregnancy, obesity, chronic diseases, and medications, have shown an effect on the incidence of CTS [2]. Bad habits, such as increased daily smartphone addiction, were found to increase the incidence of CTS [3]. Understanding these correlations can help physicians prevent CTS and improve patient care [4].

The diagnosis of CTS depends on an evaluation of both the patient's medical history and clinical examination. The most common provocative tests now are Durkan's test, Phalen's test, and Tinel's sign, which have various degrees of sensitivity and specificity [5]. The sensitivity and specificity range from 0.47 to 0.84 and 0.11 to 0.56, respectively [5]. The most provocative sensitivity test was Phalen’s test (68%), and the most specific was Durkan's test (77%). Treatment of CTS is based on its severity; for mild and moderate-severity cases of CTS, electrotherapy, splinting, physiotherapy, and corticosteroid injection showed their efficacy with various degrees of safety and effectiveness [1]. An MRI is a highly accurate diagnostic approach for CTS, measuring the circumferential surface area, flattening ratio, relative intensity of the median nerve signal, and bowing of the retinaculum [6]. Electrodiagnostic studies (EDX) are superior in diagnosing CTS, indicating damage to the median nerve, and detecting muscle impairment innervated by the median nerve. Electrodiagnostic studies have high accuracy, sensitivity, and specificity, up to 87% and 27%, respectively. It could be used to diagnose CTS, identify the severity, or follow up on the progress after surgery. A summary of different diagnostic tests is presented in Table 1. Cases of severe CTS according to the Bland classification that show motor latency > 4.5 ms with absent sensory response have shown better response to surgical lines of treatment [6]. The main principle of surgical intervention in CTS is to decrease the pressure or decompress the tension from the median nerve passing through the carpal tunnel by cutting a small incision through the skin over the wrist deep down through the flexor tendon retinaculum. Two main approaches for this procedure are conventional open-release and endoscopic-release approaches. Gurpınar et al. carried out a study on patients who complained of CTS and haven’t shown a favorable response to surgical lines of treatment [7]. They reported comparable outcomes in both groups, although the endoscopic group had the advantages of endoscopic procedures by returning to normal life quicker and the advantage of a small incision [7].

**Table 1:** Diagnostic tests for carpal tunnel syndrome

Test	Sensitivity	Specificity
Tinel’s sign	55%	75%
Phalen’s test	68%	71%
Durkan’s test	62%	77%
Hand elevation test	85%	95%
Upper-limb neurodynamic test	71%	28%
Electrodiagnostic studies	87%	27%
Ultrasound examination	96.9%	93.6%

Recent studies have reported new approaches for surgical intervention in severe CTS cases and cases not responding to conservative treatment like thread ultrasound-guided carpal tunnel release (TCTR). In this study, we intend to summarize the recently published studies discussing surgical interventions for CTS.

## Review

Materials and methods

We searched different databases (Google Scholar, PubMed, and Scopus) up to December 2023 for relevant studies discussing available surgical interventions used in the treatment of CTS using terms like “carpal tunnel syndrome," “median nerve entrapment," ”surgical intervention,” and "open carpal tunnel release (OCTR)," etc. Each topic of the paper was searched independently for the following: (1) risk factors for CTS; (2) diagnostic tests for CTS and grading; (3) treatment options for each grade; and (4) surgical interventions used till now in treating indicated cases.

Management of carpal tunnel syndrome 

Treatment strategy changes according to the patient's condition, with mild and moderate cases responding to conservative treatments such as physical therapy, corticosteroid injection, splinting, shock waves, and yoga. Severe cases require surgical decompression of the carpal tunnel. A comprehensive review by Hernández-Secorún et al. in 2021 found that electrotherapy is useful when paired with splinting but not better than manual therapy. Corticosteroid injections are not more effective than saline solutions or other treatments. One 5% dextrose injection is beneficial for managing CTS symptoms. Wrist splints may supplement treatments. In the short term, manual and electrotherapy may be effective for advanced cases of CTS, and in diabetic CTS, physical therapy could be effective. However, the targeted impact of conservative therapy must be addressed [8].

Indications of surgical intervention

Surgical intervention in CTS is recommended if medical treatment fails to treat the condition or in severe cases of CTS (hand numbness, atrophic changes of the thenar and hypothenar muscles, and reduced hand activity). Before going through any treatment strategy, the primary cause of CTS should be recognized first, as the causative factor should be treated initially [9]. Moreover, CTS evaluation using different classification systems guides the management strategy (Table 2). The MRI classification introduced by Ng et al. (2020) showed that although the flattening ratio, signal intensity of the median nerve, and bowing ratio all exhibited significant differences, none of these MRI parameters was as accurate as the cross-sectional area (CSA) of the median nerve for CTS diagnosis and grading the severity [6]. A meta-analysis was published in 2018 comparing the clinical outcomes between CTS patients managed with surgical intervention and patients who were treated conservatively. The surgical intervention showed better outcomes over a longer period of six months. The symptoms in the surgical intervention group were significantly relieved compared to the conservative treatment group. It was improved by 0.52 points lower in a five-point symptom severity score (95% CI (0.27-0.78)). It also showed the superiority of the surgical intervention over treating the patients conservatively in terms of functional improvement, although there were no statistically significant results between the two arms (mean deviation (MD) = 0.06; CI 95% (0.10-0.22); I2 =84%; p<0:00001) [10].

**Table 2:** Classification criteria of carpal tunnel syndrome based on neurophysiological abnormality (Bland’s criteria) and MRI findings CTS: carpal tunnel syndrome; DML: distal motor latency; ABP: abductor pollicis brevis (a muscle in the hand); SNAP: sensory nerve action potential; APB: abductor pollicis brevis (referring to the electrical activity measurement from this muscle); mm²: square millimeters, a unit of area measurement used in the MRI criteria to represent the circumferential surface area of the wrist; ms: milliseconds, a unit of time measurement used to assess nerve conduction velocities and latencies; MV: millivolts, a unit of electric potential used to measure the amplitude of motor and sensory potentials; MRI: magnetic resonance imaging, a diagnostic technique that uses magnetic fields and radio waves to produce detailed images of the body’s internal structures

Classification	Mild	Moderate	Severe
Bland criteria	Grade 0 (normal): patients with no neurophysiological abnormalities	Grade 3 (moderate): patients with sensory potential preserved and complaining of motor slowing; DML to ABP< 6.5 ms	Grade 4 (severe): CTS patients (motor terminal latency > 4.5 ms and < 6.5 ms with absent SNAP)
			Grade 5 (very severe CTS): patients with motor terminal latency > 6.5 ms
	Grade 1 (very mild): very mild symptoms and the cases were detected only by two sensitivity tests		
			Grade 6 (extremely severe): CTS patients with surface motor potential from APB < 0.2 mV, peak-to-peak
	Grade 2 (mild): in patients with sensory nerve conduction velocity was slow on the finger and had normal terminal motor latency		
MRI criteria	In wrists showing circumferential surface area > 15 mm^2^		In wrists showing circumferential surface area > 19 mm2.

Different approaches to surgical intervention

In 1924, Herbert Galloway was the first to report the surgical treatment of CTS. Surgical methods have become prevalent since then. This is thought to be the most effective treatment, with many options studied and proposed. The main dubiety is the choice between minimally invasive short wrist incisions, endoscopic approaches, or standard open techniques (longitudinal incision at the wrist with direct visibility of the flexor retinaculum) that can all be used to do decompression surgery with the current development of ultrasound [11].

Open approaches

Conventional Open Surgery

The contents of the carpal tunnel are released surgically by transecting the flexor retinaculum. Surgeons could use either a standard or a mini-incision. The classic incision's borders are the radial border of the hypothenar muscle, and then the incision extends until we reach the distal crease of the wrist. Five centimeters is the standard length of the CTS incision, but we can extend it as required for a better examination. A 2 cm transverse incision on the ulnar side of the wrist stripes or a longitudinal incision starting from the mid-palm and finishing at the area proximal to the palm are the mini-incision procedures. The main disadvantages of the latter two approaches are higher intricacy, scar discomfort, and an increased risk of partial release of the transverse ligament. Other approaches, like multiple small incisions or slightly modified longitudinal incisions, have shown promising outcomes. Numerous studies have shown that all mini-incision techniques are safe and effective [7,12].

Following a surgical skin incision, the surgeon should visualize the flexor retinaculum by going through the fat and fascia of the palm. When properly exposed, it should be entirely divided longitudinally, with median nerve-relieved tension and visualization. The final surgical stages are wound closure and wound dressing. Making an incision of OCTR by a little incision utilizing a group of specifically developed instruments while keeping the benefits of following up on the condition directly and avoiding problems of dangerous injury to essential tissues. The instruments are made of a metal guide with a central groove for an angled knife holder [13]. The palm scar following the OCTR incision could alter the hand's size and appearance. Also, nerve damage could happen during the surgery as there is the palmar nerve branch of the median nerve, which starts at the palmar distal region of the forearm and separates into two branches before traveling superficially to the transverse carpal ligament (TCL) of the hand, could be injured during the exposure of the procedure.

The recurrent motor nerve, a branch of the median nerve, that ordinarily supplies the thenar muscles, is another nerve that could be damaged. Its injury could have a substantial impact on thumb function [14,15]. Due to the preferable outcomes of the OCTR, more technical advancements are being conducted in this technique. A study conducted by Atthakomol et al. in 2022 compared minimally invasive surgery (MIS) with toolkits and short incisions. The short incision was about a 3 cm incision distal to the flexion crease at the mid-palm area of the wrist. Then a scissor dissection deep into subcutaneous fat and skin was performed to divide the palmar fascia under direct vision. The MIS with the tool kit was applied by making a 1.5-2 cm incision along the radial axis of the ring finger, about 2 cm apart from the wrist crease. After dissecting the palmar and subcutaneous aponeurosis, a working space was created which was visualized by a tube inserted in the retinaculum area just on top of the transverse carpal ligament. Smooth and complete separation of the TCL at the proximal and distal portions by the MIS-CTS cut knife was achieved. All the procedures were directly visualized by the MIS-CTS tube. The study concluded that there is no difference between the two techniques due to the comparable steps of the procedures. But considering the cost-effectiveness, the short-term technique would have the upper hand, while for patients concerned with the cosmetic appearance, the MIS-CTS would be a better choice because of the short incision scar [16].

Reconstruction of the Transverse Carpal Ligament (RTCL)

Patients with CTS who underwent the typical procedure of OCTR complained of the incidence of pillar pain, scar sensitivity, or grip weakness and recovery. The etiology of adverse effects connected to carpal tunnel release (CTR) is vague, but biomechanical changes from flexor retinaculum (FR) disruption may be responsible [17]. A meta-analysis by Lai et al. (2019) compared surgical carpal tunnel release with flexor retinaculum reconstruction (FRR) and surgical CTR only. Long-term functional status was noticed to be improved in the surgical release with FRR. Anyhow, no significant difference was noted between both groups who combined the surgical decompression with FRR and those who used the surgical decompression only regarding the improvement of grip strength and symptom relief in short and long-term follow-up [18]. A randomized clinical trial was conducted by Gutiérrez-Monclus et al. on 117 cases of severe idiopathic CTS. The study assessed transverse carpal ligament reconstruction by comparing two groups: the intervention group performed open retinaculum release followed by a reconstruction of the TCL, while the control group performed only retinaculum release. Combining open release with TCL repair leads to a considerable improvement in the strength of the grip over the medium term. On the other hand, open release alone reduced symptom severity in patients who complained only of unilateral idiopathic, severe CTS [19].

Minimally invasive surgical approaches

Endoscopic Carpal Tunnel Release (ECTR)

The first ECTR with a single and dual-portal approach was reported and introduced by Okutsu et al. [20], who began employing endoscopy to achieve carpal ligament release. Several other authors, including Agee et al [21], have used and adapted this technique over the years. At the proximal wrist striatum, Agee used a portal to enter between the flexor carpi ulnaris and the palmaris longus. Following soft tissue dissection, the palmaris longus tendon was exposed. The endoscope was then inserted via the portal into the transverse ligament from the ulnar side, and the ligament was severed from distal to proximal [21]. The fundamental limitation of this method is the poor visibility of the nerves and vessels passing [21,22].

Modified Endoscopic Carpal Tunnel Release (MECTR)

In 2020, Chen et al. described a new technique that could be applied and propagated in areas with poor health systems. In MECTR, the skin is incised about 1 cm in the area proximal to the transverse carpal crease and the ulnar side of the palmaris longus side, and we could extend it if needed. This new technique, compared to OCTR, showed a decreased risk of developing trauma, better safety, and is more effective and feasible to be performed in low-level healthcare facilities. For its simplicity and short learning curve, it should be more widespread in CTS management [23].

Ultrasound-Guided Carpal Tunnel Release

With the progressive development of ultrasound, surface structures can now be thoroughly investigated. This could be applied to hand and wrist joints. This includes scanning the relevant structures in at least two orthogonal planes and conducting dynamic and Doppler evaluations. Anisotropy aids the sonographer in distinguishing between the flexor tendons and the median nerve in the carpal tunnel. Using the Doppler mode, we can localize the ulnar and radial arteries. Ultrasound can be used to investigate the anatomy of the components of the carpal tunnel and its boundaries [24]. Ultrasound-guided CTR with smaller incisions was found to reduce surgery-related morbidity and speed up recovery. This procedure has numerous advantages for the patient, the hospital, and society [18]. The surgical process is brief, and it can be conducted in an outpatient ambulatory setting, boosting patient turnover. Furthermore, the recovery time for returning to work is only seven days. Rojo-Manaute et al. (2016) described and documented the efficacy of ultra minimally invasive US-guided CTR (UMIU-CTR) with retrograde TCL release utilizing a hook knife. The phrase "ultra-minimally invasive" alludes to the procedure's little tissue damage: a 1 mm incision port and the retention of the fascial layer directly palmar to the TCL. Ultra-minimally invasive CTR leads to rapid functional recovery, fewer postoperative complications, and equivalent neurologic healing as mini-open CTR in people with symptomatic primary CTS [25].

Comparison between different surgical techniques

A study conducted by Orhurhu et al. in 2020 on patients with CTS compared the outcomes of ECTR and OCTR. It found that both endoscopic and open methods resulted in significant postoperative pain reduction, although in long-term follow-up, pain scores after ECTR and OCTR procedures were the same, even after the transcendency of ECTR in postoperative pain scores over open procedures directly after the surgery. Endoscopic surgery provides much more pain alleviation, typically in the early period after the surgery. The meta-analysis reported a statistically significant decrease in the time needed to recover with ECTR; the ECTR group returned to their daily activities 10 days earlier than the OCTR group (MD = -9.56; 95% CI -12.51 to -6.60) [26]. Another systematic review compares ECTR and also evaluates the superiority of the endoscopic techniques of either single-portal or two-portal endoscopy [26]. Without considering the number of portals, it shows that ECTR had a considerably higher risk of transitory postoperative nerve injury than open release, even though overall complications and the need for second operations were comparable.

Dual-portal endoscopic release dramatically reduces scar tenderness when compared to single-portal and open procedures. The frequencies of symptom alleviation and the satisfaction of the patient were not substantially different across treatment groups [27]. A study conducted by Zeng et al. (2023) on 40 patients with 40 wrists compared the clinical outcomes of mini-open surgery vs. ultrasound-guided needle release with corticosteroid injection in patients with CTS [28]. The study demonstrated that before and three months after treatment, it appraised the Boston Carpal Tunnel Questionnaire (BCTQ), electrophysiological parameters (sensory conduction, velocity, distal motor latency, and sensory nerve action potential of the median nerve), and ultrasound parameters (flattening ratio, cross-sectional area, and transverse carpal ligament thicknesses) [28]. The total treatment duration, healing time, cost, and complications for the two groups were also noted. Each group had a significant difference in the electrophysiological results of the BCTQ. The BCTQ and ultrasound results were obtained preoperatively and three months postoperatively (P < 0.05). In both groups, no incidence of infection, bleeding, vascular, nerve, or tendon injuries was noticed.

Both mini-open surgery and ultrasound-guided needle release are effective treatments for CTS cases. When compared to mini-open surgery, ultrasound-guided needle release in combination with corticosteroid injection results in a smaller incision, lower cost, shorter treatment time, and a faster return to daily activities [28]. A study was conducted in 2023 by Zheng et al. to assess the new technique of MECTR by reporting the outcomes compared to the traditional OCTR. Twenty-two cases treated with this technique reported no significant difference in the BCTQ, assessing the severity and functionality of patients with carpal tunnels in two weeks, one month, and three months after operation [29]. A large retrospective cohort study was conducted on 4,338 patients undergoing open release, or ECTR, with a one-year follow-up to assess long-term results. A rate of 2.08% of ECTR cases required revision of carpal tunnel release, while the open-release group required revision in 0.71% of cases. The revision of the release was also found to be related to other variables like male sex, tobacco use, concurrent cubital tunnel syndrome, and diabetes [30].

Safety evaluation of different surgical interventions

Regarding the safety of the available surgical interventions for CTS, a study conducted by Vasiliadis et al. [31] in 2015 reported some outcomes regarding the safety of both OCTR and ECTR. An overall population of 878 in the ECTR group and 806 in the OCTR group were recorded in the 15 studies that reported the incidence of recurrent CTS. It reported 24 recurrences in the ECTR group and 19 cases in the OCTR group (odds ratio (OR) 1.02; 95% CI 0.55 to 1.90). Eleven studies included in this systematic review reported that 28 cases required re-operation from a total of 1,596 operations which included 20 patients in the ECTR group and eight in the OCTR group, from a total population of 869 in the ECTR group and 727 in the OCTR group. Conversion from endoscopic to open release was performed in 15 cases. Due to intraoperative difficulties in three cases, re-operations were encouraged by the injury of the superficial palmar branch, which happened in one case in the ECTR group and two cases in the OCTR group. In total, 12 cases in the ECTR group and 12 cases in the OCTR group experienced major complications from a total population of 1366 in the ECTR arm and 1199 in the OCTR arm.

Out of the total 2,442 wrists included in the study, 63 cases in the ECTR group and 120 cases in the OCTR group complained of minor complications out of the 1,275 wrists in the ECTR arm and 1,167 wrists in the OCTR arm. Endoscopic carpal tunnel release had a lower risk of developing minor complications in comparison with OCTR (OR 0.50; 95% CI 0.31, 0.82) [31]. Another study reported some outcomes regarding the safety of the interventions. Postoperative complications in the ECTR group were pillar pain (n = 6), numbness or tingling (n = 13), and persistence of symptoms (n = 6). While in OCTR, the reported complications were reflex sympathetic dystrophy (n = 7), pillar pain (n = 9), and hypertrophic/painful scar (n = 5). Four cases experienced the need for re-operation in the ECTR group, and three events of re-operation were noticed in the OCTR group [26]. As pain is one of the postoperative outcomes, a study conducted by Schroeder et al. in 2022 on 1,125 patients with CTS who underwent open and endoscopic surgery found that postoperative opioid use was similar in both groups [32]. In a study conducted by Liawrungrueang et al. in 2022, they found a decrease in short-term pain in the ECTR in a follow-up period extended to two weeks [33]. Modified ECTR showed a significantly shorter operation time of 33.57 ± 4.21 minutes but no difference in relieving scar pain during the follow-up period [29]. The largest study comparing the most common techniques, ECTR and OCTR, was conducted on 735,631 patients with CTS undergoing surgical release after the 1:1 match between the two groups of ECTR and OCTR, which included 292,626 cases. Various complications and results assessing the safety of each intervention were reported. The OCTR group experienced a higher incidence of wound complications (OR 1.97, 95% CI 1.74-2.23) and readmission at 30 days (OR 1.89, 95% CI 1.73-2.06). In addition to that, the high reimbursement cost of ECTR ($310.60 ± $1639.57, P<0.001) is considered a disadvantage of this technique, but considering the overall superiority of ECTR, it could be considered worth the financial charges [23,34].

Recommendations for future research

The debate over the superiority of different surgical techniques for treating CTS has been ongoing for decades. Even though many studies have proven ECTR to be superior in terms of key pinch strengths, satisfaction rates, faster healing times, and fewer scar-related problems, there is still a dearth of high-quality evidence. This is concerning, as clear evidence is often provided for prevalent diseases and related management options. The decision between open-release or endoscopic approach surgery is usually left to the surgeon, depending on their experience and preference along with the patients' related factors. A simple algorithm that allows surgeons to choose the method based on their experience and preferences, along with patient factors, has shown the best results. Yet, when deciding between these techniques, the odds of transient nerve injury should be considered. Relevant authors often strongly recommend their proposed technique; comprehensive systematic reviews and meta-analyses reveal that relevant biases and constraints prevent standardization and do not give sufficient evidence of a technique's advantage over others. Surgeons should prioritize minimally invasive releases, which allow for greater neurovascular structure visualization and longer-term symptom alleviation. A high-level evidence method is required, which should separately study specific topics and provide details of every single treatment option. An observational cohort should be large enough, bias should be minimized, and current heterogeneity should be overcome. In conclusion, while all surgical approaches have sufficient reliable results and have been proven effective, there is still a lack of universally accepted standardization.

## Conclusions

Releasing tension over the median nerve is the primary goal of surgical intervention in CTS management. Thus multiple surgical approaches have been developed over time to minimize the side effects of surgery. Open carpal tunnel release offers proper visualization, although it has multiple complications such as prolonged recovery time and improper healing, which can lead to sensitive scars. Meanwhile, while uniportal and double-port ECTR decrease surgical incision length and shorten post-surgery recovery time, they have a limited surgical view. Additionally, other approaches have been developed to improve safety, such as using an ultrasound-guided approach or combining OCTR with reconstruction of the flexor retinaculum or transverse ligament. In conclusion, the best surgical approach depends on the patient's case and the surgeon's experience.
